# A rare presentation of Kikuchi Disease with Hemolytic Uremic Syndrome

**DOI:** 10.12669/pjms.35.2.735

**Published:** 2019

**Authors:** Salwa Tauseeq Khan, Rubina Naqvi, Rahma Rashid, Sana Abbas Naqvi

**Affiliations:** 1 *Salwa Tauseeq Khan, MBBS. Department of Nephrology, Sindh Institute of Urology and Transplantation, Karachi, Pakistan*; 2 *Rubina Naqvi, MBBS, MD (Nephrology). Department of Nephrology, Sindh Institute of Urology and Transplantation, Karachi, Pakistan*; 3 *Rahma Rashid, MBBS, FCPS (Pathology). Department of Histopathology, Sindh Institute of Urology and Transplantation, Karachi, Pakistan*; 4 *Sana Abbas Naqvi, MBBS. Department of Nephrology, Sindh Institute of Urology and Transplantation, Karachi, Pakistan*

**Keywords:** Kikuchi Disease (KD), Hemolytic Uremic Syndrome (HUS), Acute Kidney Injury (AKI)

## Abstract

Kikuchi disease (KD) or also known as Kikuchi Fujimoto disease is named after scientists Kikuchi and Fujimoto who describe the disease in Japan in 1972. KD originally reported from Asia but later case reports from different regions of world have been published. It is a benign condition of necrotizing histiocytic lymphadenitis which mimic like Lymphoma, diagnosis of KD is based on histo-pathological findings from lymphnodes. It is a rare condition and mostly case reports have been published, it can have an association with other pathologies. We aim to report a case where KD has been found in a young woman in association with hemolytic uremic syndrome and acute kidney injury.

## INTRODUCTION

Kikuchi disease (KD) or also known as Kikuchi Fujimoto disease is named after scientists Kikuchi and Fujimoto who describe the disease in Japan in 1972.^1^ This disease has been reported from all over the world but more from Asia. It is a benign condition of necrotizing histiocytic lymphadenitis which mimic like Lymphoma, diagnosis of Kikuchi Fujimoto disease is based on histo-pathological findings from lymphnodes.^1^

## CASE REPORT

We report a case of a thirty year old woman, married, mother of three, and resident of a village which is located 560 KM from Karachi (the city where this institution is located). The lady gave birth to a child 12 days prior to her admission in this hospital. The neonate was alive, born preterm, through spontaneous vaginal mode. The child was born at home with assistance of local women; reportedly there was no unusual blood loss at time of child birth. The woman did not had any antenatal visits therefore blood pressure recording and urinary analysis not available. She became anuric after child birth, thus referred to this hospital which is a tertiary renal care unit.

When reached for further details regarding her illness it was found that she had non specific poly arthralgias and undocumented intermittent low grade fever over last approximately two months. There was no history of decline in weight or loss of appetite, patient had no previous history of Tuberculosis or of contact with tuberculosis patients. She had a history of taking analgesics for non specific joint and body aches. There was no history of any other medical problem or surgical procedure in past.

On arrival here her clinical examination revealed anemia, no peripheral edema, multiple palpable non tender right sided cervical lymph nodes, normal nails and skin. Her blood pressure was 130/70, temperature 100^0^F, pulse 100/minute and respiratory rate 22/minute. Cardiovascular, respiratory, abdominal and neurological examination was normal.

Laboratory hematological parameters were as follows; hemoglobin was 7.0 g/dl (reference range 12.0-15.5), white blood cell count was 18.0*×*10^9^/L (reference range, 3.5–10.5*×*10^9^/L) and consisted of 77% neutrophils, 8% monocytes, 13% lymphocytes, 1% basophils, and 1% eosinophils. Platelets were 738,000 (reference range 150,000-400,000 *×*10^9^/L), ESR was 65 mm during first hour.

Routine chemistry included urea of 225 mg% (reference range 10-50 mg%), creatinine was 12.8 mg% (reference range 0.5-1 mg%), serum sodium was 145 mEq/l (136-149), potassium 5.6 meq/L (3.5-5.2), chloride 100 mEq/L (98-107), bicarbonate 22 mEq/L (25-29). LDH was 612 (reference range 91-180 U/L), liver function tests, serum calcium and total proteins were within normal limits. Serology revealed C3 of 0.7 (reference range 0.79-1.52 g/L), C4 of 0.4 (reference range 0.16- 0.38 g/L), ANA and Anti DNA were negative. Viral serology for HBV, HCV, EBV and HIV were negative. Urinalysis on dipstick revealed protein 3+ and rest normal. Microbiology for blood and urine cultures was negative. Chest radiograph was negative for any masses or lymphadenopathy. Ultrasonography of abdomen showed normal size kidneys and normal rest of examination.

**Fig.1:**
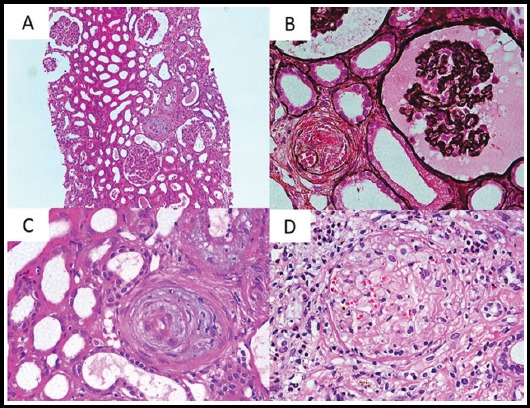
Histologic findings of renal biopsy. A. Medium power view showing ischemic wrinkling of capillary tufts, marked mucinous intimal thickening and obliteration of lumena of the arterioles (H&E, ×200). B. High power view showing ischemic wrinkling of capillary tufts and arteriole showing endothelial cell swelling, lumenal obliteration and extravasation of RBCs into the wall. (JMS, ×400). C. High power view showing marked mucinous intimal thickening, endothelial cell swelling and almost complete obliteration of lumen (H&E, ×400). D. High power view showing completely infarcted glomerulus with acellular closure of capillary lumena and fragmentation of RBCs. (H&E, ×400)

**Fig.2:**
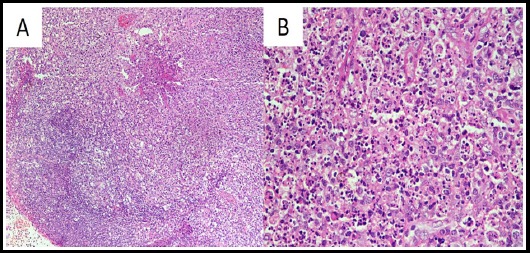
Histologic findings of cervical lymph node. A. Medium power view of lymph node showing partial effacement of nodal architecture with paracortical expansion (H&E, ×200). B. High power view of lymph node (paracortical area) showing sheets of histiocytes alongwith few eosinophils and florid karyorrhectic debris. (H&E, ×400)

Her renal biopsy was performed which revealed findings consistent with HUS, and cervical lymph node biopsy revealed findings of KD. (Fig 1 and 2)Informed consent was taken before all procedures, that is vascath placements and lymph node or renal biopsy. These consent forms include one segment mentioning that this information can be shared in scientific publications without mentioning patient’s identification.

The patient was treated with renal replacement in form of hemodialysis. While plasma exchanges done for 10 consecutive days, after getting renal biopsy report. Oral prednisolone @ 1mg/kg/day was started after receiving lymph node biopsy report. Patient was discharged because she was desperate to go home to see her children, as her renal functions did not improved till time of discharge from hospital she was advised to come back in three days but she never returned. When contacts was made on cell number provided at time of hospitalization, patient’s brother informed that she died two days after reaching home, it was sudden death and they could not consult even nearby doctor.

## DISCUSSION

In literature KD has been reported in association with systemic lupus erythematosis (SLE), Epstein Bar virus (EBV), hemophagocytosis with a common presentation of fever and lymphadenopathy.^2^-^4^ The pathogenesis of Kikuchi disease in unclear but histological changes suggests immune response with presence of T cells and exposure to infectious agents with histiocytes. A definite diagnosis of Kikuchi disease can be made on biopsy taken from lymph node which reveals lymphocytes (predominantly T cells), histiocytes and macrophages. Histiocytes and macrophages contain phagocytized debris from degenerated lymphocytes.^1^-^7^ Absence of granuloma differentiates it from tuberculosis.

Atypical hemolytic uremic syndrome (aHUS) is a complement-mediated disease characterized by hemolytic anemia, thrombocytopenia and acute kidney injury. Pregnancy associated aHUS refers towards the thrombotic microangiopathy resulting from uncontrolled complement activation during pregnancy or the postpartum period. This is a devastating disease which has a limited clinical understanding and treatment options.^8^ Classically pregnancy associated aHUS occurs in post partum period, the overall prevalence reported is 26%, with 71% presenting during the first pregnancy.^9^ Long term renal prognosis in pregnancy associated aHUS has been reported poor.^8^,^9^ Even the response to plasma exchange has also been reported poor in pregnancy related HUS.^8^

To best of our knowledge based on literature search no case has been reported with Kikuchi disease along with Hemolytic uremic syndrome and renal failure. Our patient had two pathologies and there is no way to find out whether aHUS was in association of KD or it was a separate entity pregnancy associated aHUS. She gave birth to her child pre maturely and had some symptoms that were probably related to KD. We have seen many patients with pregnancy associated aHUS (unpublished data) and renal recovery in these women is not always very good, some respond to plasma exchanges while others even after timely reaching to this facility, do not recover. We have no experience with use of Eculizumab in these patients, as it is not available at our pharmacy because of its high cost (at this institution all medical aid provided to patient free of cost and institution is funded from state budget and large part from philanthropists). In this particular patient renal recovery has not yet been started and she was allowed to visit home and children just to avoid added psychological burdens, her cause of death is uncertain and may be hypothesized from any cause ranging from electrolyte disturbance, acid base disorder, thrombo-embolic phenomenon to sudden cardiac event. According to literature although Kikuchi disease is self limiting in most cases, the prognosis is different according to the underlying cause.

## CONCLUSION

Overall it is clear that Kikuchi disease should be kept in mind as a differential diagnosis when a patient comes with fever and lymphadenopathy even in the tuberculosis endemic countries. The Kikuchi disease is rare, benign and treatable when timely diagnosed. Also this can have association with other pathologies occurring at same time.

### Author’s Contribution

**STK:** Identified the case, plotted the initial manuscript and searched literature

**RN:** Reviewed manuscript initial draft, made desirable changes, did further literature addition and finalized.

**RR:** Processed both lymph node and renal biopsies and established histo pathological diagnosis, provided figures for manuscript.

**SAN:** Helped first author in history taking, physical examination of patient and initial drafting
